# Microarrays for global expression constructed with a low redundancy set of 27,500 sequenced cDNAs representing an array of developmental stages and physiological conditions of the soybean plant

**DOI:** 10.1186/1471-2164-5-73

**Published:** 2004-09-29

**Authors:** Lila O Vodkin, Anupama Khanna, Robin Shealy, Steven J Clough, Delkin Orlando Gonzalez, Reena Philip, Gracia Zabala, Françoise Thibaud-Nissen, Mark Sidarous, Martina V Strömvik, Elizabeth Shoop, Christina Schmidt, Ernest Retzel, John Erpelding, Randy C Shoemaker, Alicia M Rodriguez-Huete, Joseph C Polacco, Virginia Coryell, Paul Keim, George Gong, Lei Liu, Jose Pardinas, Peter Schweitzer

**Affiliations:** 1 Department of Crop Sciences, University of Illinois, Urbana, IL, 61801, USA; 2 Center for Computational Genomics and Bioinformatics, University of Minnesota, Minneapolis, MN, 55455, USA; 3 USDA/ARS, Department of Agronomy, Iowa State University, Ames, IA, 50011, USA; 4 Department of Biochemistry, University of Missouri, Columbia, MO 65211, USA; 5 Department of Biology, Northern Arizona University, Flagstaff, AZ, 86011, USA; 6 Keck Center for Comparative and Functional Genomics, University of Illinois, Urbana, IL, 61801, USA; 7 Epicentre, 726 Post Road, Madison, WI, 53713, USA; 8 USDA/ARS, National Soybean Research Laboratory, University of Illinois, Urbana, IL, 61801, USA; 9 Food and Drug Administration, Rockeville, MD, 20850, USA; 10 The Institute for Genome Research, 9212 Medical Center Drive, Rockville, MD, 20850, USA; 11 Department of Plant Science, McGill University, 2111 Lakeshore, St. Anne-de-Bellevue, QC, H9X3V9, Canada; 12 Mathematics and Computer Science, Macalester College, St. Paul, MN, 55105, USA; 13 Department of Microbiology, School of Medicine, University of Nevada-Reno, Reno, NV, USA; 14 Biotechnology Resource Center, Cornell University, Ithaca, NY, 14853, USA

## Abstract

**Background:**

Microarrays are an important tool with which to examine coordinated gene expression. Soybean (*Glycine max*) is one of the most economically valuable crop species in the world food supply. In order to accelerate both gene discovery as well as hypothesis-driven research in soybean, global expression resources needed to be developed. The applications of microarray for determining patterns of expression in different tissues or during conditional treatments by dual labeling of the mRNAs are unlimited. In addition, discovery of the molecular basis of traits through examination of naturally occurring variation in hundreds of mutant lines could be enhanced by the construction and use of soybean cDNA microarrays.

**Results:**

We report the construction and analysis of a low redundancy 'unigene' set of 27,513 clones that represent a variety of soybean cDNA libraries made from a wide array of source tissue and organ systems, developmental stages, and stress or pathogen-challenged plants.

The set was assembled from the 5' sequence data of the cDNA clones using cluster analysis programs. The selected clones were then physically reracked and sequenced at the 3' end. In order to increase gene discovery from immature cotyledon libraries that contain abundant mRNAs representing storage protein gene families, we utilized a high density filter normalization approach to preferentially select more weakly expressed cDNAs. All 27,513 cDNA inserts were amplified by polymerase chain reaction. The amplified products, along with some repetitively spotted control or 'choice' clones, were used to produce three 9,728-element microarrays that have been used to examine tissue specific gene expression and global expression in mutant isolines.

**Conclusions:**

Global expression studies will be greatly aided by the availability of the sequence-validated and low redundancy cDNA sets described in this report. These cDNAs and ESTs represent a wide array of developmental stages and physiological conditions of the soybean plant. We also demonstrate that the quality of the data from the soybean cDNA microarrays is sufficiently reliable to examine isogenic lines that differ with respect to a mutant phenotype and thereby to define a small list of candidate genes potentially encoding or modulated by the mutant phenotype.

## Background

Genes of higher plants are expressed in a coordinated fashion during development of tissue and organ systems and in response to different environmental conditions. This regulation may be tightly linked for some sets of genes, for example, in a specific biochemical pathway. Expression of regulatory genes may modulate the expression of key genes or entire sets of genes in individual pathways. The investigation of single gene expression patterns as determined by RNA blotting or quantitative reverse transcriptase PCR have been used to understand how different temporal, developmental, and physiological processes affect gene expression. With recent advances in genomics, very large numbers of genes can now be simultaneously analyzed for their expression levels in a comparative fashion between two biological states using microarray or biochip technology. Several techniques for the 'global' analysis of gene expression have been described [1-4]. These include (a) high density expression arrays of cDNAs on conventional nylon filters with radioactive probing; (b) microarrays or 'chips' using fluorescent probes, and (c) serial analysis of gene expression (SAGE).

Methods for global expression analysis require either knowledge of the entire genome of an organism or accumulation of a large EST (expressed sequence tag) database for the organism. In soybean (*Glycine max*), more than 286,000 5' EST sequences have been generated and deposited in public databases [[5], and this report]. These 5'ESTs represent a collection of 80 cDNA libraries from different tissue and organ systems at various stages of development and under diverse physiological conditions. Collaborative, multidisciplinary research to enhance the development of plant genome resources and information that would be publicly available for gene expression, gene tagging, and mapping has been a priority in recent years in plants of agronomic importance, including soybean [6]. Here, we report the development, qualification, and use of 27,513 members of a low redundancy set of tentatively unique cDNAs or 'unigenes' in soybean. The 3' sequence of this set was determined and microarrays constructed. The public availability of the low redundancy clone set, sequence information, and microarrays reported here will greatly enhance gene discovery and genomic scale research in soybean and other legumes by the community of researchers. For example, we illustrate the use of the 5' and 3' sequence-verified cDNA microarrays to determine organ-specific expression and we demonstrate their potential to discover the molecular basis of specific mutations in closely related isogenic lines.

## Results and discussion

### Cluster analysis of 280,000 ESTs reveals 61,127 'unigenes' in soybean

**Figure 1:**
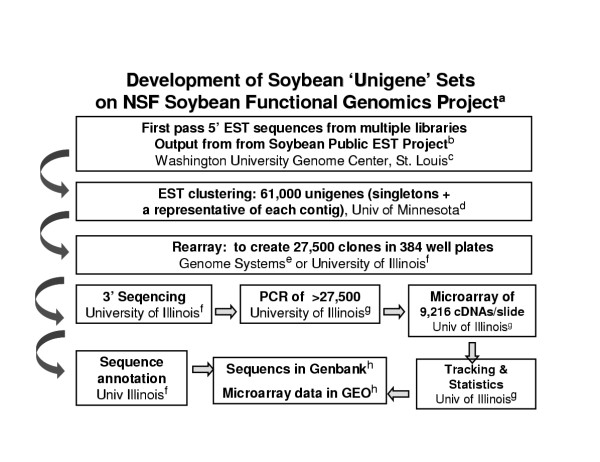
Steps in the construction and documentation of cDNA microarrays using a low redundancy soybean 'unigene' set of 27,513 cDNA clones. See Methods for details. (a) NSF Plant Genome Program project "A Functional Genomics Program for Soybean" (NSF DBI #9872565); (b) Soybean Public EST project [5]; (c) Washington University Genome Center, St. Louis, MO; (d) Center for Computational Genomics and Bioinformatics, University of Minnesota, Minneapolis, MN [20]; (e) Genome Systems, St. Louis, MO, until its closure; (f) Keck Center for Comparative and Functional Genomics, University of Illinois, Urbana, IL [21] (g) Soybean Functional Genomics, Department of Crop Sciences, University of Illinois, Urbana, IL [30]; (h) databases maintained by the National Center for Biotechnology Information, Bethesda, MD [22].

Figure 1 illustrates an overview of the data generation and analysis used to create a low redundancy 'unigene' set and its use in construction of cDNA microarrays. The 5' EST sequence information was used as the raw material including the addition of over 30 new cDNA libraries and more than 160,000 5' sequences since an initial report [5]. In total now, over 80 libraries and over 280,000 5' EST have been generated from many tissue and organ systems of various stages of development, ranging from roots, shoots, leaves, stems, pods, cotyledons, germinating shoot tips, flower meristems, tissue culture derived embryos, and pathogen challenged tissues. These libraries, with the one exception of library Gm-r1030 described below, were non-normalized. Thus, the mRNAs that are more abundant in various tissue and organ systems will be more highly represented in the EST collection. To remove redundancy and identify unique sequences, the ESTs were assembled using the Phrap program [7] into contiguous regions (contigs) representing overlapping sequences based on EST sequence similarity. In this way, longer overlapping sequences of expressed genes are assembled. Identical sequences that represent redundant mRNAs of various sizes are also recognized and the number of sequences in a contig in a non-normalized cDNA library is a rough approximation of the relative abundance of that particular mRNA within that tissue.

The combined number of contigs and singletons (sequences that occur only once) resulting from a computer assembly of ESTs is an estimate of the number of unique genes in the organism. As the number of ESTs grows, the number of unique genes in the organism will continue to be refined. Our current contig analysis of the entire public EST collection for soybean of 286,868 sequences yields 61,127 'unigenes' of which 36,357 are contigs and 24,770 are singletons.

The finding of 61,127 soybean unigenes by EST cluster analysis agrees well with an independent contig and unigene assembly in the databases of The Institute for Genomic Research (TIGR) which shows 30,084 contigs and 37,601 singletons for a total of 67,826 tentatively unique sequences from among 334,730 sequences representing all publicly available sequences clustered in Release 11 [8]. Other large scale plant EST collections as analyzed by the TIGR gene indices [8] show 42,301 unigenes for *Arabidopsis thaliana *(of 247,429 ESTs), 36,976 for *Medicago truncatula *(of 189,919 ESTs), 31,012 for tomato (of 156,645 ESTs), 109,509 for wheat (of 494,195 ESTs) and 56,364 for maize (of 377,188 ESTs). The complete genome sequence for Arabidopsis has revealed an estimated 26,000 genes [9]. Of course, the unigene sets determined by EST clustering are only estimates of the number of unique genes in an organism and depend on the number of ESTs available, the technologies used to make the libraries, and the bioinformatic methods used to assign clusters. The soybean genome is approximately 1.2 × 10^9 ^bp which is about 7.5 times the size of the Arabidopsis genome and twice that of tomato, but less than half the size of the maize genome. Thus, it is not unexpected that soybean may have a larger number of unigene clusters than Arabidopsis or tomato, for example. Although soybean is not hexaploid in origin as is modern wheat, it is thought to be an ancient autotetraploid and many examples of duplicate loci exist in soybean.

### Virtual subtraction using high density cDNA filter arrays increases gene discovery in immature cotyledon libraries that abundantly express storage protein gene transcripts

**Figure 2:**
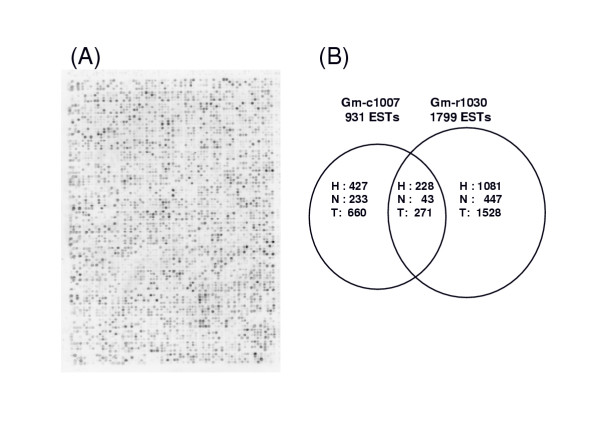
Gene discovery is increased by selection of weakly expressed cDNAs clones from a cDNA library made from immature cotyledons. (A) Phosphorimager pattern: A high density membrane containing 18,432 double spotted colonies from the Gm-c1007 cDNA library made from immature cotyledons was hybridized with ^33^P-labeled cDNAs transcribed from mRNAs isolated from immature cotyledons. (B) Graphical representation of the new cDNAs selected by the normalization process using filter hybridization. Circles represent the 931 total sequences obtained from the non-normalized source cDNA library Gm-c1007 versus the 1799 sequences of Gm-r1030 that were selected as weakly expressed sequences from the filter hybridization experiments shown in part (A). The intersection of the circles represent sequences common to both sets. H = number of sequences with hits in the databases; N = number of sequences that did not have a hit in the databases; and T = total number of sequences.

Certain tissues contain large amounts of specialized transcripts. Developing soybean cotyledons, for example, contain large amounts of RNA transcripts representing highly expressed storage protein genes. In order to select for some of the weakly expressed cDNA clones from a mid-maturation stage cotyledon library, we used a virtual subtraction approach using high densisty filters. A total of 18,000 bacterial clones containing cDNAs from library Gm-c1007 (immature cotyledons of 100–300 mg fresh weigh range from Williams variety) was printed in high density on nylon filters and probed with ^33^P-cDNA produced by reverse transcription of total mRNA isolated from immature soybean cotyledons. Figure 2A shows the highly complex hybridization pattern resulting from using total mRNA from immature cotyledons to probe the high density filter. The intensity of each dot represents the hybridization signal and the relative abundance of that cDNA in the message population. The phosphorimager pattern was quantified by image analysis software and 5000 of the lowest expressing clones were selected. The cDNA clones were physically reracked to a new set of 384 well plates to form the filter-normalized reracked library designated Gm-r1030. The 5' end of these clones were then sequenced at Washington University. Figure 2B shows that 1,528, or 85%, of the 1799 sequences within the filter-driven reracked library Gm-r1030 were novel and not found in any of the 931 sequenced clones from the Gm-c1007 source cDNA library. Thus, the filter normalization method was an effective way to identify cDNAs with low expression and increase gene discovery in libraries that contain large numbers of transcripts from highly expressed genes. The virtual subtraction method using high density cDNA filters compares favorably with other mRNA subtraction methods used to create normalized libraries during the cloning process [10].

### Selection and 3' sequencing of 27,513 soybean cDNAs from the soybean unigene set to use in microarrays

High density cDNA arrays of bacterial cultures spotted on nylon membranes and probed with radioactively labeled transcripts are useful for gene discovery as illustrated in Figure 2 above, but they have limited use for quantifying the relative abundance of transcripts expressed in independent mRNA samples. An alternative method to the high density filters is microarray technology [2,3] in which PCR-amplified cDNA inserts, or oligonucleotides, are printed on glass slides and probed with mRNAs populations that have been separately labeled with different fluorescent probes.

To enable global expression studies, the ideal would be to have each gene represented at least once on an array. Toward this aim, we selected 27,513 of the cDNA clones by four successive clustering assemblies of the soybean cDNAs performed as the 5' EST data accumulated. Table 1 shows the four reracked sets of cDNA clones (Gm-r1021, Gm-r1070, Gm-r1083, and Gm-r1088) and the level of uniqueness within them as defined by Phrap and CAP3 analysis [7,11]. Thus, the clustering was conducted periodically as the number of 5' EST input sequences grew in size. After each clustering, the previously selected and reracked cDNAs were excluded from subsequent reracking lists. In order to develop a 'unigene' set for soybean, a single representative of each contig was chosen. To select a representative from each contig, we chose the cDNA clone corresponding to the EST that was found at the furthest 5' region of each contig. Thus, we are selecting the cDNA clones most likely to be near full-length.

**Table 1:** Comparison of the percent unique sequences as determined by either CAP3 or Phrap analysis for the 5' and 3' ESTs represented in each of the four successive reracked clone subsets that constitute the low redundancy soybean 'unigene' set

Rerack order & name	Number cDNAs	No. ESTs clustered^a^	Cap3^b^	Phrap^b^	% Unique ESTs^c ^Cap3 or Phrap
1. Gm-r1021	4,089	2,797, 5'	2,202 s 259 c	2,054 s 334 c	88.0% : 80.4%
1. Gm-r1021	4,089	2,797, 3'	1,836 s 413 c	1,682 s 505 c	85.4% : 78.2%
2. Gm-r1070	9,216	6,938 5'	5,566 s 620 c	5,116 s 831 c	89.2% : 78.0%
2. Gm-r1070	9,216	6,938 3'	4,284 s 1,124 c	3,900 s 1,340 c	85.7% : 75.5%
3. Gm-r1083	4,992	3,879 5'	3,426 s 200 c	3,289 s 260 c	93.5% : 79.7%
3. Gm-r1083	4,992	3,879 3'	2,474 s 599 c	2,256 s 723 c	91.5% : 76.8%
4. Gm-r1088	9,216	7,434 5'	6,295 s 521 c	5,909 s 745 c	91.7% : 89.5%
4. Gm-r1088	9,216	7,434 3'	4,719 s 1,173 c	4,152 s 1,513 c	79.3% : 76.2%
Entire set, 1–4	27,513	27,513 5^d^	21,873 s 2,402 c	18,663 s 3,966 c	88.2% : 81.2%
Entire set, 1–4	27,513	21,048 3'	11,959 s 4,156 c	8,341 s 5,641 c	73.0% : 63.3%

^a ^Unless otherwise noted, the ESTs included in the cluster analysis represent only the cDNAs for which both the 5' and 3' sequences are known and for which the read length is over 200 bases.

^b ^The number of singletons (s) and number of contigs (c) are shown.

^c ^The % unique sequences is the number of singletons plus the number of contigs divided by the total number of ESTs.

^d ^In this analysis, all 5' sequences were included even if the corresponding 3' sequence was not known.

EST clustering will overestimate the number of unique genes as some of the shorter ESTs will not overlap and thus are falsely counted as independent, unique sequences. However, the clustering analysis can also falsely lump non-identical members of gene families into the same contig based on conservation of sequence similarity in the coding region. The 3' sequencing is especially useful for resolving both of these issues as there is generally more variation in the 3' UTR in plant genes than in the coding region. For those reasons and as a quality control of the reracking process, we sequenced the 3' end of the reracked cDNAs. From the 27,513 total 3' sequencing attempts on the tentatively unique cDNAs represented in Table 1, a total of 22,088 sequences met the criteria of high quality sequence. The 3' sequencing was more problematic than the 5' sequencing due to termination of the sequencing reactions at some of the long polyA tails characteristic of soybean and many other plant cDNAs. An anchored primer was used to increase the success rate (see Methods). The average length of the 3' ESTs was 526 bases compared to the average 5' sequence read length of 474 for 280,094 ESTs.

Since the clustering analyses were performed at successive intervals as the EST collection grew in size, we repeated the Phrap contig analyses separately using only the input sequences of each cDNA rerack for which both a 5' and 3' EST were known. We also performed a CAP3 analysis [11]. Table 1 shows that CAP3 values for the 5' sequence yielded 88.0 to 93.5% unique sequences while the Phrap values were slightly lower at 78.0 to 89.5% unique sequences. Interestingly, the estimate of unique sequences using the 5' EST data did not change substantially from reracked library r1021 where only approximately 6800 ESTs were clustered through library r1088 where over 250,000 sequences were clustered. A separate cluster analysis of only the 27,513 input 5' sequences revealed 81.2 to 88.2% unique sequences by Phrap and CAP3 analyses, respectively.

The 3' ESTs were also separately subjected to CAP3 or Phrap analysis. The CAP3 values showed a slightly higher level of uniqueness (or lower level of redundancy) with 79.3 to 91.5% for CAP3 in the successive clustering analyses versus 75.5 to 78.2% unique sequences as determined by Phrap. An overall figure of 73.0% for the CAP3 analysis on the 21,048 total 3' sequences clustered was found versus 63.3% for Phrap.

The differences between the 5' and 3' levels of uniqueness (i.e., 88.2% versus 73.0% for the entire sets as determined by CAP3) can be explained by the nature of reverse transcriptase action. The reverse transcriptase was primed using an oligo dT primer and so the cDNAs will begin at the 3' end and will terminate randomly at variable sites as the enzyme progresses to the 5' end of the mRNA template. Thus, 5' ESTs often begin at variable sites. Therefore, even though two 5' ESTs may have originated from the same mRNA transcript, they will not cluster if they are non-overlapping and will be counted as two separate ESTs. The 3' soybean EST reads begin just after the poly A tail and produce longer average read lengths than the 5' soybean ESTs; thus, the 3' ESTs are more likely to form an overlapping contig if there is any redundancy among them.

The 5' and 3' sequence (where known) of each soybean unigene were queried against the non-redundant (nr) database with BLASTX [12]. Annotations were assigned to each 5'and 3' if the best match had an e value of ≤10^-6^. Table 2 shows a complete cross list of all identifiers for each member of the 27,513 soybean unigenes in the reracked libraries including the 5' and 3' annotations.

**Table 2:** *Information contained in a comprehensive cross list of soybean unigene clone IDs. *Shown are various identifiers and annotations for 27,513 reracked cDNAs used in microarray construction. The full list is provided with arrays and available upon request.

Cross List Identifiers (for each cDNA clone)	Example (one of 27,543 cDNAs)	Comments
Reracked Clone ID	Gm-r1021-12	The individual cDNA clone ID in the 384-well destination plates after reracking or rearraying of the selected clones from the cDNA source library plates.
Reracked Plate ID	Gm-r1021 #1	The 384-well reracked plate name in increments of 384 (ie., 1, 385, 769, etc.)
Reracked row_column position	A12	Position of the clone in the 384-well reracked plate
Reracked 3' Keck Sequence ID	GM210001A21A6	Sequence identifier assigned by the Keck Center for the 3' EST
Reracked 3' Genbank Accession	AW348131	Genbank assigned accession number for the 3' EST
Reracked 3' Annotation	glutathione S-transferase GST 22 [Glycine max]	Top BLASTX hit for the 3' EST, at E10^-6 ^or lower
Source Clone ID	Gm-c1004-464	The individual cDNA clone ID in the 384-well source plate.
Source Plate ID	Gm-c1004 #385	The 384-well source plate name in increments of 384 (ie., 1, 385, 769, etc.)
Source row_column position	D8	Position of the clone in the 384-well source plate
Source WashU Sequence ID	sa26h04.y1	Sequence identifier assigned by Washington University, 5' EST
Source 5' Genbank Accession	AI442436	Genbank assigned accession number for the 5' EST
Source 5' Annotation	glutathione S-transferase GST 22 [Glycine max]	Top BLASTX hit for the 5' EST at E10-6 or lower
Source Library	Gm-c1004	Name of the cDNA source library
Cultivar/Genotype	Williams	Specific information on the soybean variety or genotype
Tissue/Developmental Stage	Entire roots of 8-day old seedlings	Tissue/organ system/stage from which the cDNA library was constructed

### Construction of microarrays representing the 27,513 soybean unigene cDNAs

The current 'unigene' collection (or tentatively unique sequences) represents low redundancy sets of cDNA clones. We have processed all of these cDNAs for microarrays, as outlined in the Methods section, into three sets of 9,216 cDNAs per array. As shown in Table 3, these include reracked libraries Gm-r1070 (a set of 9,216 cDNA clones from libraries of various developmental stages of immature cotyledons, flowers, pods, and seed coats); Gm-r1021 plus Gm-r1083 (a set of approximately 9,216 cDNA clones from 8-day old seedling roots, seedling roots inoculated with *Bradyrhozobium japonicum, *2-month old roots, and whole seedlings); and Gm-r1088 (a collection of 9,216 cDNA clones from a number of libraries made from cotyledons and hypocotyls of germinating seedlings and leaves and other plant parts subjected to various pathogens or environmental stress conditions and also from tissue-culture derived somatic embryos). As an example, the Gm-r1070 set contains 3,938 tentatively unique cDNAs that are directly derived from two flower cDNA libraries (Gm-c1015 and Gm-c1016) that were sequenced deeply with over 14,000 5' ESTs obtained from these two libraries. In addition, a total of 2,639 cDNAs on the array are directly derived from source libraries made from the immature stages of cotyledon development and representing over 11,000 input sequences from the cotyledon stages of seed development.

**Table 3:** Soybean microarrays and low redundancy and low redudancy unigene sets built from the public EST collection

Microarrays and Reracked Unigene cDNA sets ^a^	Source cDNA Library ^a^	No. of cDNAs on array	Soybean Variety	Soybean tissues ^b^
***Set 1. ****Gm-r1070: 9216 cDNAs highly representative of developing seeds and flowers*				
Gm-r1070	Gm-c1016	2242	Williams 82	immature flowers
"	Gm-c1015	1696	Williams 82	mature flowers
"	Gm-c1008	869	Williams	whole young pods (2 cm)
"	Gm-c1029	589	Williams	immature cotyledons from 25–50 mg fresh weight seed
"	Gm-c1010	234	Williams	immature cotyledons 100–200 mg seed fresh wt.
"	Gm-c1011	88	Williams	immature cotyledons 100–200 mg seed fresh wt.
"	Gm-c1007	528	Williams	immature cotyledons 100–300 mg seed fresh wt.
"	Gm-c1030	1200	Williams	immature cotyledons 100–300 mg seed fresh wt. low expressing cDNAs fromGm-c1007 filter hybridizations
"	Gm-c1023	89	T157	immature seed coats from seed of 100–200 mg fresh wt.
"	Gm-c1019	1681	Williams	immature seed coats from seed of 200–300 mg fresh wt.
**Set 2. ***Gm-r1021+Gm-r1083: 9216 cDNAs highly representative of roots*				
Gm-r1021(c)	Gm-c1004	4224	Williams	roots of 8-days old seedlings
Gm-r1083	Gm-c1009	1117	Williams	roots, 2 month old plants
"	Gm-c1028	3055	Supernod	roots innoculated with B. japonicum
"	Gm-c1013	820	Williams	whole 2–3 week old seedlings
**Set 3. ***Gm-r1088: 9216 cDNAs highly representative of seedlings, leaves, and stressed or pathogen challended tissues*				
Gm-r1088	Gm-c1019	426	Williams	immature seed coats from seed of 200–300 mg fresh wt.
"	Gm-c1023	929	T157	immature seed coats from seed of 100–200 mg fresh wt.
"	Gm-c1027	2706	Williams	cotyledons of 3- and 7-day-old seedlings
"	Gm-c1036	613	Jack	somatic embryos cultured on MSD 20 for 2 to 9 mo.
"	Gm-c1075	304	Jack	differentiating somatic embryos cultured on MSM6AC
"	Gm-c1064	707	Williams	epicotyl, 2 week old seedling, auxin treatment
"	Gm-c1065	1309	Williams	germinating shoot, cold stressed, 3 day old seedlings
"	Gm-c1066	191	Williams	leaf and shoot tip, salt stressed, 2 wk. old seedling
"	Gm-c1067	438	Williams82	germinating shoot, 3 day old seedling, auxin treatment
"	Gm-c1068	630	Williams82	leaf, drought stressed. 1 month old plants
"	Gm-c1072	365	PI 567.374	leaves and shots from 2–3 week old seedlings induced for SDS symptoms
"	Gm-c1073	324	Williams 82	leaves and shoots from 2–3 week old seedlings included for SDS symptoms
"	Gm-c1074	274	Williams 82	9–11 day old seedlings induced for HR response by P. syringae carrying avrB gene

^a^ More description of the reracked and source libraries are available in Genbank at

^b^ Tissues were collected from plants grown in greehouse or growth chamber except for the immature and mature flowers which were collected from plants grown in the field.

^c^ Since the Gm-r1021 reracked liabrary contains 4089 cDNAs, a total of 135 were repeated to obtain an even 9216 when combined with the Gm-r1083 cDNAs.

The cDNAs from the sequence-driven, reracked clone sets were amplified by PCR using the Qiagen-purified cDNA templates that were prepared for 3' sequencing (as opposed to amplification of the inserts directly from *E. coli *cultures containing the plasmid DNA). All 27,513 PCR reactions were performed with generic M13 forward and reverse primers using a robotic pipettor. Approximately 25% of the purified PCR cDNA inserts were subjected to agarose gel electrophoresis for quality control. Of these, the average insert size was estimated to be 1,340 bp for library Gm-r1021, 1,110 bp for library Gm-r1070, 1,259 for library Gm-r1083, and 1,269 bp for library Gm-r1088.

The 9,216 amplified inserts of each set were singly spotted onto glass slides as outlined in the Methods section. A set of 64 control or 'choice' clones was assembled by hand into one 96-well plate (designated Gm-b10BB) and printed eight times repetitively throughout each array. Thus, the total number of spots on the array is 9,728 consisting of 9,216 cDNAs from the unigene set plus 512 (64 cDNAs × 8 repeats) from the choice clones. The choice clones were selected for various reasons. Some represent constitutively expressed genes (such as ubiquitin and EF1). Some are cDNAs whose expression is restricted to a subset of specific plant tissues (such as Rubisco or seed storage proteins). Some are clones of enzymes representing commonly used antibiotic resistance markers in transgenic plants (as hygromycin or kanamycin resistance), and 32 are cDNAs that represent at least 13 different enzymes of the flavonoid pathway. The flavonoid pathway was chosen because the corresponding genes often respond to many biotic and abiotic stress conditions and it has been widely studied in plant systems.

### Soybean microarrays have potential to reveal the molecular basis of a mutant phenotype

**Figure 3:**
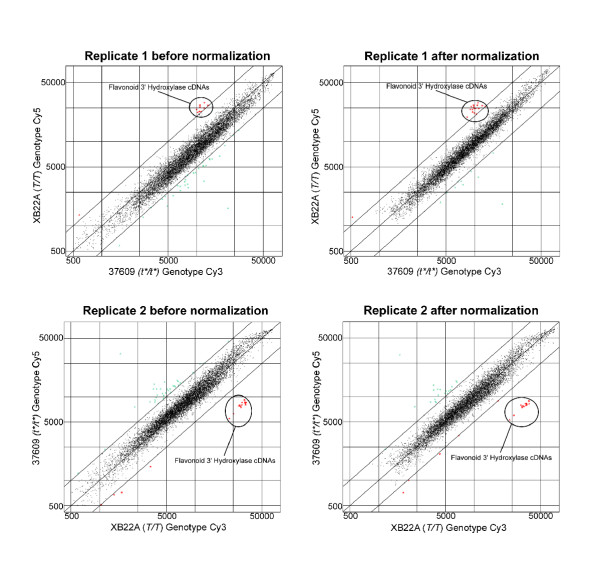
The scatter plots of the log values of expression data from two duplicate microarray slides before (left) and after flagging and normalization (right). RNAs were extracted from seed coats of the 50–75 mg per seed fresh weight range by standard methods [13]. Replicate 1 was hybridized with Cy5 labeled RNA from seed coats of the *T*/*T *genotype and Cy3 labeled RNA from seed coats of the isogenic t*/t* mutant line. Replicate 2 is a dye swap experiment in which the mRNA from the *T*/*T *genotype is labeled with Cy3 and the isogenic t*/t* line is labeled with Cy5. The lines in each graph indicate expression either two-fold higher or two-fold lower than equivalent levels of expression. The dots encircled by the box represent repeats of flavonoid 3' hydroxylase cDNAs on the array that are overexpressed in the RNA samples from seed coats of the *T*/*T *genotype.

Figure 3 illustrates an example of the reliability of the soybean microarray approach using dual labeled RNA probes from two near isogenic lines. These data illustrate the potential to discover novel genes by analysis of contrasting probes from mutant and normal lines. In this experiment, we compared two isogenic soybean lines that differ only at the *T *locus. The *T *locus controls the color of the pigment in the trichome hairs on the stems, leaves, and pods of the plant and also modifies the composition of the flavonoids and the color of the seed coats. Total RNA extracted from developing seed coats of line XB22A (*T*/*T *genotype) was labeled with Cy3 and compared to RNA extracted from the same stage of developing seed coats from an isoline containing the spontaneous mutation 37609 (t*/t* genotype) that was labeled with Cy5. A replicate experiment with a dye swap was also performed. Hybridizations were performed to microarrays constructed with the low redundancy set Gm-r1070 representing cDNAs from seeds, seed coats, and flowers. Figure 3 shows both replicates before and after the flagging and normalization procedures conducted as described in the Methods section. As shown in Figure 3, the normalization procedure serves to compensate within slide differences between the Cy3 and Cy5 intensity levels to shift the majority of spots to the line of equivalent expression between the isogenic lines. Also, as shown in Figure 3, very few of the 9,728 cDNAs on the array were reproducibly found to be expressed differentially in the two genotypes at levels higher or lower level than two-fold. A group of 16 of these (encircled by a line) are overexpressed in the *T*/*T *line relative to the t*/t* line by approximately three-fold. These cDNAs correspond to the flavonoid 3' hydroxylase cDNAs that were repetitively spotted on the array as members of the 'choice' clone set. Table 4 shows the Cy5 and Cy3 intensities and the ratios of the two replicate slides for all cDNA spots that exceeded a two-fold difference after normalizations within each slide and between the replicate slides. Only 23 cDNAs were found to have values that meet the criteria of exceeding a two-fold differences in both of the replicate slides. Of these 16 were the repetitively spotted flavonoid 3' hydroxylase cDNAs. Only an additional 22 cDNAs (known as partial hits) had values exceeding two-fold levels in one but not both of the slides replicates. Thus, out of over 9200 cDNAs represented on the array, there are relatively few that show differential expression between the RNAs in the normal and mutant lines.

**Table 4:** Differentially expressed cDNAs detected in dual labeling microarray experiments comparing isogenic lines of the T locus in soybean.

Clone ID	Genbank	Intensities^a^				Expression Ratios			Functional Annotation^d^
	3' Accession	Replicate 1		Replicate 2					
			
		XB22A (*T*/*T*)	37609 (t*/t*)	XB22A (*T*/*T*)	37609 (t*/t*)	XB22A/37609 (*T*/*T*) / (t*/t*)			
			
		Cy 5	Cy3	Cy3	Cy5	Rep 1^b^	Rep 2^b^	Ave^b,c^	

*Overexpressed in XB22A*									
Gm-b10BB-23	AF499730	28686	10847	38350	8194	2.645	4.680	3.520	Flavonoid-3' hydroxylase
Gm-b10BB-23	AF499730	26794	9746	34956	7839	2.749	4.459	3.512	Flavonoid-3' hydroxylase
Gm-b10BB-22	AF499731	23979	9018	33272	7626	2.659	4.363	3.440	Flavonoid-3' hydroxylase
Gm-b10BB-23	AF499730	26094	9231	23580	5264	2.827	4.479	3.427	Flavonoid-3' hydroxylase
Gm-b10BB-23	AF499730	26812	10670	35600	7963	2.513	4.471	3.350	Flavonoid-3' hydroxylase
Gm-b10BB-22	AF499731	25663	9746	32520	7685	2.633	4.232	3.338	Flavonoid-3' hydroxylase
Gm-b10BB-22	AF499731	24020	9468	34329	8241	2.537	4.166	3.295	Flavonoid-3' hydroxylase
Gm-b10BB-22	AF499731	24578	10218	35465	8158	2.405	4.347	3.267	Flavonoid-3' hydroxylase
Gm-b10BB-23	AF499730	24662	9850	26041	5957	2.504	4.371	3.208	Flavonoid-3' hydroxylase
Gm-b10BB-22	AF499731	22240	9526	31641	7643	2.335	4.140	3.138	Flavonoid-3' hydroxylase
Gm-b10BB-22	AF499731	24548	11084	35328	8233	2.215	4.291	3.100	Flavonoid-3' hydroxylase
Gm-b10BB-22	AF499731	19465	8343	30897	7912	2.333	3.905	3.098	Flavonoid-3' hydroxylase
Gm-b10BB-23	AF499730	26572	12004	37720	8844	2.214	4.265	3.084	Flavonoid-3' hydroxylase
Gm-b10BB-23	AF499730	21583	9921	31828	7410	2.175	4.295	3.082	Flavonoid-3' hydroxylase
Gm-b10BB-22	AF499731	21122	10431	37273	8852	2.025	4.211	3.028	Flavonoid-3' hydroxylase
Gm-b10BB-23	AF499730	26053	12897	37691	8164	2.020	4.617	3.027	Flavonoid-3' hydroxylase
*Underexpressed in XB22A*									
Gm-r1070-484	BE819850	2843	6235	3931	8702	0.456	0.452	0.454	Bowman-Birk inhibitor
Gm-r1070-8083	BE823467	2471	5070	3498	8557	0.487	0.409	0.438	Ribonucleoprotein homolog
Gm-r1070-8006	BE823540	3353	7237	4145	12219	0.463	0.339	0.385	Trypsin inhibitor, Kunitz
Gm-r1070-9195	BE824378	1889	4384	2383	7562	0.431	0.315	0.358	No hits found
Gm-r1070-120	BE657237	3083	7548	3769	11877	0.408	0.317	0.353	Trypsin inhibitor, Kunitz
Gm-r1070-8909	BE824331	3771	10945	3756	13568	0.345	0.277	0.307	Beta conglycinin
Gm-r1070-9099	BE824364	1806	19569	1663	31399	0.092	0.053	0.068	Albumin precursor/leginsulin

(a) Intensities after background subtraction and global normalization between replicates and within each slide are shown.

(b) The mean ratios of the 16 flavonoid hydroxylase cDNAs are significant below the p = 0.0001 level in a t-test compared to 2.0 as the mean.

(c) The average ratio of both slides is calculated by as follows: (XB22A Rep 1 intensity + XB22A Rep2 intensity) / (37609 Rep 1 intensity + 37609 Rep 2 intensity)

(d) The matches for all of the functional annotations were to soybean (*Glycine max*) sequences except for the ribonucleoprotein homolog which was to *Arabidopsis thaliana*.

An examination of the ratios of the 16 repetitively spotted flavonoid 3' hydroxylase cDNAs using a *t *test showed that the mean ratio of the repeated cDNAs on replicate 1 (2.424) were statistically significant at a P value of 0.0001 when compared to an expected mean of a 2.0, or a two-fold expression difference. The low P values were also found for replicate 2 and for the mean value (3.245) of both replicates. Thus, the flavonoid 3' hydroxylase cDNAs are statistically significant outliers in the microarray analysis.

The microarray data presented here and showing that the cytoplasmic levels of the flavonoid 3' hydroxylase are higher in the *T*/*T *line agree very well with RNA blot data which showed that the flavonoid 3' hydroxylase gene has reduced expression in the seed coats of the t*/t* isoline compared to the *T*/*T *lines [13]. In addition to the RNA blot data showing differences in these mutant lines, we have definitively shown that the flavonoid 3' hydroxylase is encoded by the *T *locus by sequence data of other alleles of the locus and by genetic cosegregation data [13]. We do not know the reason for the change in the expression levels of the seven other cDNAs as shown in Table 4, most of them representing various seed or storage type proteins. While the *T *locus does determine the flavonoid and pigment compounds synthesized in various tissues including seed coats and trichomes, it is possible that the flavonoid compounds themselves modulate an additional effect on seed protein synthesis in the seed coats. Alternatively, the observed differences in the levels of these cDNAs could be due to an artifact during the dissection procedure. We know from Northern blots, that flavonoid 3' hydroxylase is highly expressed in the seed coats, but is not expressed in the cotyledons so any small amount of contaminating cotyledon cells due to imprecise dissection of the seed coats of one line versus the other could lead to observed differences in seed protein RNAs.

As this example in Figure 3 and Table 4 illustrates, the use of dual labeled mRNAs from near isogenic lines to probe microarrays is a powerful approach with which to obtain a small list of candidate genes from among the thousands examined by microarray analysis. In this example only eight functionally different cDNAs (or seven if the two trypsin inhibitor cDNAs are counted as one) of over 9200 cDNAs spotted on the array met the criteria of exceeding two-fold levels of expression in both replicates. If a cDNA is repetitively spotted on an array, as were the flavonoid 3' hydroxylase cDNAs, then the data are statistically significant. After identifying a short list of candidate genes, it is then feasible to test them by other methods (as RNA blotting, quantitative RT-PCR, RFLP or SNP analysis) in order to find an association of a particular cDNA with the mutant phenotype. Of course, if a particular mutation has a regulatory or epigenetic effect on a large number of downstream RNAs, or if a mutation does not affect the abundance of an mRNA, then the global expression approach may not be effective in identifying the primary nature of the mutant locus. For example, the standard recessive *t *allele at the *T *locus is the result of a premature stop codon and does not affect abundance of the flavonoid 3' hydroxylase mRNA to the same extent as does the t* mutation at that locus [13].

### Tissue specific gene expression using the soybean microarrays

**Figure 4:**
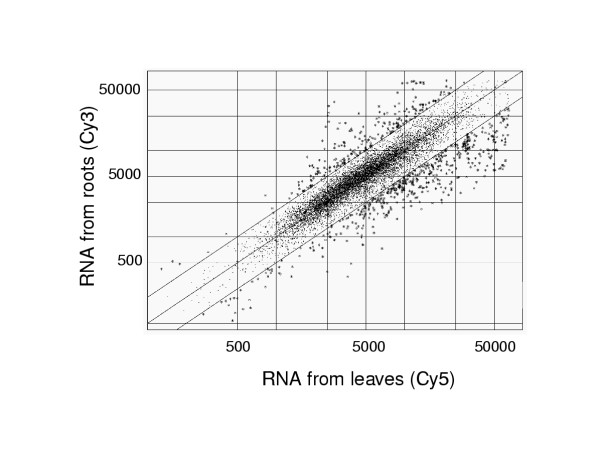
One of the scatter plots of the log values of expression data from microarray slides hybridized with Cy3 labeled RNA from leaves and Cy5 labeled RNA from roots. Many cDNAs have differential expression above or below the two-fold level as indicated by the lines.

In contrast to the results with near isogenic lines of the *T *locus which showed that relatively few cDNAs showed differential expression between the two very closely related lines, the soybean microarrays reveal larger numbers of cDNAs showing differential mRNA abundance levels in different tissue types of the same plants. For illustration, Figure 4 shows one of the two replicates of a dual labeling experiment using the low redundancy set Gm-r1088 of 9,216 cDNAs. The Cy3 labeled probe in this experiment was RNA from roots of hydroponically grown soybean plants, and the Cy5 probe was RNA from leaves of the same plants. The upper ratio threshold is 2.0 and the lower threshold is 0.5.

Table 5 lists a selection of the 300 clones with significantly elevated expression in leaves (ratios below the 0.5 threshold) and the 125 clones with significantly elevated expression in roots (ratios above the 2.0 threshold) that consistently varied more than two-fold in both of the replicated slides. Among the clones with elevated expression in roots are chalcone isomerase, putative aquaporins (tonoplast intrinsic protein), tubulin, an auxin-repressed protein, peroxidase, sucrose synthase and PEP carboxylase. These plants were not inoculated with *Rhizobia *and therefore only a few nodulin-related genes were observed to be markedly upregulated: nodulin-26, nod factor binding lectin-nucleotide phosphohydrolase (GS50, Accession # AF207687) and a MtN19 homolog.

**Table 5:** A selection of genes that are differentially expressed in leaves or in roots

Clone Identification	GenBank accession no.	Average Ratio^1^	Function^2^	Annotation (BLAST hit and organism)
*Leaves up-regulated*				
Gm-b10BB-41	AI495218	0.103	en	Rubisco (*Glycine max*)
Gm-r1088-7900	BU550654	0.294	en	Light harvesting chlorophyll a/b binding protein (*Arabidopsis thaliana*)
Gm-r1088-8981	BU549821	0.261	en	Photosystem I subunit (*Oryza sativa*)
Gm-r1088-3538	BU546899	0.365	en	Thylakoid lumen protein (*Arabidopsis thaliana*)
Gm-b10BB-47	AW185639	0.171	en	Plastocyanin precursor (*Glycine max*)
Gm-r1088-2905	BU546179	0.258	en	Trehalose-6-phosphate phosphatase (*Arabidopsis thaliana*)
Gm-r1088-7106	BU548940	0.150	st	Vegetative Storage Protein (*Glycine max*)
Gm-r1088-5827	BU548964	0.465	df	Acidic chitinase (*Glycine max*)
Gm-r1088-6724	BU549206	0.217	cmg	Putative calreticulin (*Oryza sativa*)
Gm-r1088-3756	BU546067	0.415	cmg	Cytochrome P450 (*Pyrus communis*)
Gm-r1088-8229	BU550097	0.454	cmg	Catalase (*Glycine max*)
Gm-r1088-5243	BU547961	0.143	cmg	Putative serine carboxypeptidase II-3 precursor (*Oryza sativa*)
Gm-r1088-1433	BU545435	0.200	cmg	H protein (*Flaveria anomala*)
Gm-b10BB-12	AI900038	0.211	cmg	F3H (Flavanone-3-Hydroxylase) (*Glycine max*)
Gm-r1088-4994	BU547986	0.205	cmg	Matrix metalloproteinase MMP2 (*Glycine max*)
Gm-r1088-2794	BU547254	0.138	cmg	Putative lipoic acid synthase (LIP1) (*Arabidopsis thaliana*)
Gm-r1088-4578	BU547484	0.165	cmg	Lipid transfer protein-like protein (*Retama raetam*)
*Roots up-regulated*				
Gm-r1088-5321	BU547784	3.806	no	Nodulin-26 (*Glycine max*)
Gm-r1088-7410	BU550525	2.304	no	MtN19 homolog (*Medicago truncatula*)
Gm-r1088-6955	BU551008	4.630	no	Similar to nodulins and lipase homolog (*Arabidopsis thaliana*)
Gm-r1088-6384	BU550458	4.431	to	bZIP transcription factor (*Arabidopsis thaliana*)
Gm-b10BB-11	AI930858	3.008	df	Chalcone isomerase (*Glycine max*)
Gm-r1088-6204	BU547671	2.541	cmg	Putative aquaporin (tonoplast intrinsic protein) (*Arabidopsis thaliana*)
Gm-r1088-2818	BU546503	2.848	cmg	Phosphoenolpyruvate carboxkinase (*Flaveria trinervia*)
Gm-r1088-1741	BU544616	2.724	cmg	Similar to sucrose synthase (*Pisum sativum*)
Gm-b10BB-37	AW309104	6.228	cmg	Proline-rich protein (*Glycine max*)
Gm-b10BB-38	AI442449	2.233	cmg	DAD-1 (Defender Against apoptopic cell Death) (*Glycine max*)
Gm-r1088-5369	BU547794	8.372	cmg	Ripening related protein (*Glycine max*)
Gm-r1088-7112	BU548943	6.661	cmg	Germin-like protein (*Phaseolus vulgaris*)
Gm-r1088-5330	BU547868	4.974	cmg	Pectinesterase (EC 3.1.1.11) precursor (*Vigna radiata*)
Gm-r1088-6104	BU549267	4.554	cmg	Asparagine synthase (glutamine-hydrolyzing) (*Glycine max*)
Gm-r1088-741	BU544257	2.455	cmg	Cationic peroxidase (*Glycine max*)
Gm-b10BB-45	AW318233	2.135	cmg	Tubulin (b chain) (*Glycine max*)
Gm-r1088-5315	BU547781	4.334	u	Specific tissue protein 1 (*Cicer arietinum*)
Gm-r1088-5332	BU547869	4.267	oth	Auxin-repressed protein (*Robinia pseudoacacia*)

^1 ^The average ratios of individual values from two slides after normalization and using a dye swap procedure.

^2 ^en – energy; st – storage; to – transcription; cmg – cell growth and maintenance; u – unknown; oth – other; df – defense; no – nodulation related

The soybean proline-rich protein (SbPRP1 Accession # J05208) (represented on the array by AW309104, Gm-c1019-3688) is also among the root expressed clones. SbPRP1 has been shown to be expressed preferentially in the roots [14]. A gene of interest overexpressed in roots is the DAD-1 (Defender Against apoptotic cell Death). No Rubisco or photosynthesis related genes were observed to be over expressed in the roots as would be expected for the non-green tissues.

In leaves, genes typical for green tissues are upregulated as expected. These are the photosynthesis genes (eg. Rubisco, plastocyanin precursor, chlorophyll a/b binding protein type II, trehalose-6-phosphate phosphatase, photosystem subunits and proteins, thylakoid lumen protein, light-harvesting chlorophyll a/b binding protein), and the vegetative storage proteins. Ribosomal proteins, cytochrome P450, catalase, and chitinase were also noted as overexpressed in the leaves.

Our publicly available Gm-r1021 soybean unigene subset containing 4,098 cDNAs has also been used to examine differential gene expression in roots and shoots of older soybean plants [15]. We have previously utilized microarrays containing 9,216 clones of the Gm-r1070 set (representing many cDNAs from developing seeds, seed coats, flowers, and pods) to carry out a detailed analysis of induction of somatic embryos during culture of cotyledons on auxin-containing media [16]. The resulting transcript profiles were subjected to a cluster analysis and revealed the process of reprogramming of the cotyledons cells during the induction process. The 495 cDNAs (5.3% of the cDNAs on the array) that were differentially expressed were clustered into 11 sets using a non-hierarchical method (K-means) to reveal cDNAs with similar profiles in either the adaxial or the abaxial side of the embryos from 0 to 28 days in 7 day intervals. Among other conclusions, these global expression studies indicated that auxin induces dedifferentiation of the cotyledon and provokes a surge of cDNAs involved in cell division and oxidative burst.

Thus, the soybean cDNA arrays that we have developed from the unigene cDNA set can be used to reveal the underlying physiological and biochemical pathways potentially operative in specific tissues, developmental stages, or environmental treatments. Obviously, cDNA arrays from soybean or any other organism that are constructed with PCR inserts representing an average size of 1.1 kb will generally hybridize with any RNAs from gene family members that share greater than 85% homology. Thus, cDNA arrays will generally not distinguish expression from closely related duplicated sequences. Oligo arrays spotted with synthetic 70-mers or Affymetrix short oligo arrays have greater potential to separate the expression from close related duplicated sequences if the oligos are chosen from the 3' or 5' non-coding regions that carry more sequence variability than the protein coding regions.

## Conclusions

Although microarray data is limited from soybean and most plants other than Arabidopsis, the construction of the 27,513 member low redundancy 'unigene' cDNAs for soybean reported in this paper will greatly stimulate this area. The number of slides containing all 27,513 of the cDNAs is being reduced to one, or at most two slides, and the slides are publicly available. Spotted PCR products with average size of over 1 kb are useful not only for soybean, but for other legume species as cross hybridization to the long probes will be substantial. The 3' sequencing reported here is particularly useful for differentiating gene family members and for future design of gene specific oligo arrays of either 70-mers spotted on glass slides or by Affymetrix technology using short oligos synthesized *in situ*. The cDNA or oligo-based microarrays add to the developing suite of genome analysis approaches in soybean [17]. A few of the unlimited applications include profiling expression from genes that respond to challenges by various pathogens and by environmental stresses as drought, heat, cold, flooding, and herbicide application. Also, by analysis of the near isogenic lines of the *T *locus as an example, we demonstrated the potential of soybean cDNA arrays to be used for discovery of genes responsible for uncharacterized mutations. Future expression profiling of mutant phenotypes or of genotypes that differ in protein or oil content and other quantitative traits will yield significant clues to the genes involved in those pathways and traits.

## Methods

### Contig assembly for unigene selection

Raw sequence files of the 5' soybean EST data from Washington University or 3' data from the University of Illinois Keck Center were produced from sequence traces using the Phred base calling program [18,19]. The sequences were trimmed for leading and trailing vector and linker sequences and artifact *E. coli *sequences were removed. Quality checks included determining the number of ambiguous 'N' base calls in a sequence and trimming the leading and trailing poor quality (high-N) sections to obtain the best subsequence where the number of Ns was 4% or less of the total bases. The EST sequences were clustered into contig sets based on sequence overlap using the program Phrap [7]. The processing and analysis results for each sequence are displayed on a set of World Wide Web pages [20]. The distribution of sequence lengths in each submission set are displayed in histograms. The base call and quality information for each sequence in a submission are displayed in artificial gel images of the sequences. Each sequence is displayed as the raw sequence before vector filtering and the cleaned sequence after vector filtering. A color-coded sequence quality graph shows the part of a sequence retained after trimming as well as the regions trimmed for low quality, polyA or polyT, and vector sequences. Blast reports for each sequence are displayed and can be searched collectively for words or phrases of interest. Contig sequences and images of the contig assemblies are displayed on linked web pages along with graphs describing the contig qualities [20].

### Clone reracking and 3' sequencing

Soybean cDNA clones corresponding to the 5' most representative member of a contig or to a singleton were selected using Oracle database tables and SQL queries. The *E.coli *stocks representing those clones were reracked into new 384 well plates to form the sequence driven reracked libraries Gm-r1021 (4,089 cDNA clones), Gm-r1070 (9,216 cDNA clones) and, Gm-r1083 (4,992 cDNA clones). Initially, these were reracked from source 384-well plates to destination 384-well plates by Genome Systems (St. Louis, MO) using a Qbot and shipped on ice to the University of Illinois for extraction and 3' sequencing. Reracked library Gm-r1088 (9,216 cDNA clones) was reracked at the University of Ilinois Keck Center using a QPix robot, (Genetix, New Milton, Hampshire UK). Growth rates for the *E.coli *stocks were over 99.5%.

The cDNA libraries were all constructed in either pSPORT 1 (Invitrogen, Carlsbad, CA) or pBluesciptII SK (+) (Stratagene, La Jolla, CA) plasmid vectors in DH10B host cells. Each 384-well plate of a bacterial library was split into four 96-well, 2 ml block plates, each corresponding to a different quadrant (A1, A2, B1, B2) and grown overnight in 1 ml LB media with 100 μg/ml ampicillin. High quality DNA templates were purified using a QIAGEN BioRobot 9600 or BioRobot 8000 with QIAprep 96 Turbo miniprep kits (QIAGEN, Germantown MD). Dideoxy terminator sequencing reactions for the 3' ends of the soybean cDNA clones were conducted by the University of Illinois Keck Center for Comparative and Functional Genomics [21] using standard methods analyzed either on gel-based ABI 377 or capillary-based ABI3700 instruments. Inserts within each vector type can be sequenced from the 5' end using the M13 reverse primer and the 3' end using the M13 universal forward primer. However, for higher success rates at the 3' end, a degenerate primer consisting of [5'-TTTTTTTTTTTTTTTTTT(A/C/G)-3'] was employed in order to enhance the success of 3' sequencing reactions by eliminating the need to sequence through the poly A tail. The primer was synthesized and purified by HPLC (Qiagen Operon, Alameda CA) to remove shorter, incomplete primers. Using high quality Qiagen purified cDNA templates, the average 3' untrimmed read length was over 600 bases with a success rate of 80 to 85%. Original sequence trace files are available by ftp from the University of Illinois Keck Center [21]. The trimmed sequences were entered into Genbank [22]. The reracked 5' and 3' sequences were analyzed by both the CAP3 [11] and Phrap programs [7].

All cDNA clones of the low redundancy reracked 'unigene' sets are available to the public through Biogenetic Services, Inc., Brookings, SD, or the American Type Culture Collection, Manasas, VA.

### Annotation of the unigene cDNAs using BLASTX

The 5' and 3' sequences of the 27,513 unigene cDNAs clones were annotated using BLASTX against the nonredundant (nr) protein database with cutoff E value of 10E^-6^. The top blast hit was used as the annotation for each of the 5' and 3' ESTs represented in the unigene sets printed on the microarrays.

In some cases, the protein family assignments were also made using the Metafam program based on a BLASTX analysis against a protein sequence database consisting of a non-redundant set of sequences from SwissProt & TrEMBL [23], PIR & NRL [24], GenPept [25], and Integrated Genomics, Inc. (Chicago, IL). Each of the protein sequences in this database is also placed in a protein family in the MetaFam database [26-28]. The results from each BLASTX report were parsed and placed in an Oracle 8i database. The strong protein sequence hits from BLASTX are matched up to the MetaFam protein families to which those protein sequences belong.

### Amplification of cDNAs and preparation for use in microarray construction

All pipetting steps involved in amplifying the cDNAs by PCR, purification of the cDNAs, and assembling them into 384-well spotting plates were conducted with a Multimek TM 96 Automated pippetor (Beckman Instruments, CA) to reduce errors associated with manual pipetting.

#### Amplification

The same Qiagen plasmid DNA templates that were prepared for the 3' sequencing by a Qiagen robot at about 100+ ng/μl were also used for PCR amplification using Taq polymerase (Invitrogen, Carlsbad, CA), universal forward and reverse primers in 96 well plates using the MJ DNA Engine Tetrad (MJ Research, Waltham, MA). Four PCR reaction plates are prepared at a time, one from each quadrant of a 384-well library plate. A master mix consisting of final concentrations of 1X PCR buffer (20 mM Tris-HCl, pH 8.4, 50 mM KCl), 2 mM MgCl_2_, 0.25 mM each of dGTP, dATP, dTTP, dCTP, 1 μM of M13 universal primer, 1μM of M13 reverse primer, and 0.05 U/μl of Taq polymerase (Invitrogen, Carlsbad, CA, cat no. 18038-042) was prepared and 48 μl were aliquoted into each well of a 96 well PCR reaction plate (MJ Research MSP-9621). A 0.5 μl aliquot of an undiluted plasmid template DNA was aliquoted into the 48 μl of master mix. The plates were briefly centrifuged for 1 min at 1500 rpm and placed into an MJ PTC-200 DNA Engine for 1 min of denaturation at 94°C, and 28 cycles of 92°C for 30 sec, 56°C for 45 sec, and 72°C for 30 sec and a final extension of 72°C for 5 minutes. A typical yield from the PCR was about 30–100 ng/μl.

#### Purification

The PCR products were loaded into Millipore multiscreen plates (Millipore #MANU 03050) and were subjected to a vacuum applied at 15 psi for about 10 min until the wells were completely empty. Then 60 μl of sterile water were added to each well using the Multimek automated pipettor and the PCR products were washed. The purified products were eluted in sterile water, retrieved and then stored in 96 well plates at -20°C. A 1 μl aliquot of each well from 3 rows from each 96 well plate is run on a gel to check the quality of the PCR and purification of the cDNA. The yield after purification was between 30 and 40 μls with concentrations around 15–50 ng/μl.

#### Spotting plate assembly

The four quadrants were then reassembled into a 384-well spotting plate containing 6 μl per well: 4.5 μl of PCR product from the 96 well plates mixed with 1.5 μl of 4X Micro Spotting Solution Plus (MSP4X, Telechem, Sunnyvale, CA). Alternatively, in earlier prints, the spotting plates were assembled at a final concentration of 3X SSC, 0.01% N-lauroylsarcosine by mixing 3.5 μl of purified PCR product with 1.5 μl of 10X SSC, 0.033% Sarkosyl, pH 7.0 (1.5M NaCl, 0.15 M citric acid, trisodium salt, 1.12 mM N-lauroylsarcosine, Sigma L-9150).

### Microarray construction

A set of 9,216 prepared cDNA inserts from 24, 384-well spotting plates were single spotted onto amine coated glass slides (1 in × 3 in, Telechem Superamine, SMM slides, Telechem International, Sunnyvale, CA) using a Cartesian PyxSys 5500 robot (Genomic Solutions, Ann Arbor, MI) equipped with 16 quill pins (ChipMaker II from Telechem International) and an environmental chamber. The cDNAs were printed at 55% ± 5% relative humidity setting within the chamber and in a room that was controlled for humidity to be between 45 and 60% using room dehumidifiers as needed. Control of humidity was critical for printing.

All arrays contained 32 grids of spots arranged in an 8 × 4 matrix. Each grid had 19 rows and 16 columns of spots for a total of 9,728 spots per array. A total of 9,216 spots were the cDNAs prepared from the 'unigene' set to form 18 of the 19 rows with 288 spots per grid. After all of the 9,216 cDNAs were printed, an additional row of 16 spots was printed as the first row of each grid for a total of 32 grids × 16 spots = 512 additional spots. These cDNAs were printed from the choice clone spotting plate designated Gm-b10BB which contained 64 hand-picked clones. Thus, the 64 hand-picked, choice clones were printed 8 times each, i.e., each clone was printed in twice in four separate grids. In addition, since the Gm-r1021 library contained only 4089 cDNAs, an additional 135 were repeated in order to obtain an even 9216 cDNAs for printing when combined with the Gm-r1083 unigene set.

The three microarray platforms were entered in the Gene Expression Omnibus database [29] with platform accession numbers GPL229 for Gm-r1070, GPL1013 for Gm-r1021+Gm-r1083, and GPL1012 for Gm-r1088. Complete tables of sequence identifiers and accession numbers for the unigene cDNAs printed on arrays as illustrated in Table 2 are available [30].

### Construction of the 'choice' clone PCR plate for repetitive spotting

To construct the choice plate Gm-b10BB, 64 clones were chosen to be used as negative and positive controls for expression analysis in all microarray slides. These 64 clones were chosen to represent certain constitutively expressed genes, or other markers for particular tissues, and is also highly representative of key genes of the soybean flavonoid pathway.

The 64 clones were hand picked and grown over night in microfuge tubes containing 100 μl of YT media at 37°C, 250 rpm. The following day, microfuge tubes containing 200 μl of YT supplemented with 100 μg/ml ampicillin and 8% glycerol were inoculated with 5 μl from the previous culture and grown over night at 37°C and 250 rpm.

To create a 96 well plate of these *E.coli *stocks, 100μl of the previously grown culture were transferred to wells in columns A1 thru H8 and stored at -80°C. Wells in columns A9 thru H12 were left empty. A small database for the Gm-b10BB plate was prepared containing the name of each gene, its sequence, accession number, and the corresponding well in the Gm-b10BB plate. To make a replicate copy for sequencing, 100 μl of YT supplemented with 100 μg/ml ampicillin and 8% glycerol were inoculated with 5 μl of the -80°C *E. coli *stock and incubated overnight at 37°C and 250 rpm. Miniprep DNAs were isolated and sequenced at the University of Illinois Keck Center using a 5' M13 primer. The identity of each clone was confirmed by comparison of the sequences obtained from the Keck center with the sequences contained in our previously prepared database by using the Pairwise Blast tool available at the NCBI web page. All sequences showed >97% identity with the corresponding sequence in the database.

PCR amplification using the DNA miniprep plate as a source for templates was performed with the Mutimeck 96 automated pipetor (Beckman) as described above. All PCR products were purified and separated in 1% agarose gel to evaluate the purity of the amplified DNAs and determine their size. The purified and analyzed PCR products from the 96 well plate, Gm-b10BB, were used to assemble a 384 well spotting plate. The 384 well spotting plate contained 6 μl per well: 4.5 μl of PCR product from the 96 well plate aliquoted on each of the 4 quadrants and mixed with 1.5 μl of 4X Micro Spotting Solution Plus (MSP4X, Telechem, Sunnyvale, CA) after assembly.

### Post-print processing

After all slides were printed, the cDNAs were UV-cross linked to the slide coating with 650 m Joule ultra violet light using a StrataLinker (Stratagene, La Jolla, CA). [Note: prior to cross-linking the spots were rehydrated if necessary. Rehydration was required for the slides printed with the SSC-Sarkosyl spotting solution but was not required for those printed with Telechem spotting solution. DNA spots were rehydrated by passing the slide over a gentle vapor of steam for a few seconds until spots glistened but did not coalesce and then were quick dried on a 70°C heating block]. To remove excess spotted DNA as well as to denature attached DNA to single strands, slides were treated with the following series of washes with agitation: 2 min with 200 mls of 0.2% SDS, two 1 min water rinses, 95°C water for 2 min, 0.2% SDS for 1 min, and finally two water rinses of 1 min each. Slides were subjected to low speed centrifugation for 2 min at 500 rpm to dry and were stored in a slide rack in a dust free container.

### Plant material and RNA isolation and labelling

Seed coats and cotyledons were dissected from plants grown to maturity in soil in the greenhouse. Roots and leaves were collected from soybean plants grown for 11 days after germination in an aerated hydroponic solution with normal nutrient conditions. Total RNA was extracted using phenol-chloroform and lithium chloride precipitation methods [31,32]. RNA was further purified by use of RNeasy Mini or Maxi columns Qiagen, Valencia, CA) according to the manufacturer's instructions. Prior to labelling, the purified RNA was concentrated in a Speed Vac (Savant Instruments, Halbrook, NY) or by using YM-30 Microcon column (Millipore, Bedford, MA).

For each RNA probe, 50 to 60 μg of purified total RNA was labeled by reverse transcription in the presence of Cy3- or Cy5-dUTP [33]. Briefly: the RNA and 5μg oligo-dT 18–21 mer (Operon, Qiagen) were annealed in a 10μl volume at 70°C for 10 min and cooled on ice. A 20 μl cocktail containing 1X first strand reaction buffer, 10 mM DTT, 0.5 mM each of dATP, dCTP, dGTP, 0.2 mM dTTP, 100μM Cy3- or Cy5-dUTP (Amersham, Pharmacia) and 400 U of 200 U/μl SUPERSCRIPT™II (Invitrogen, Carlsbad, CA, cat no. #18064-014) was added to 10μl of the denatured RNA and oligo-dT mixture). The 30 μl reaction was incubated for 1 hr at 42°C, after which 200 additional units of SUPERSCRIPT™II were added and incubation was continued for another hour at 42°C. The reaction was then treated with RNAse A and RNAse H (0.5μg and 1.0 U respectively, Invitrogen, Carlsbad, CA) for 30 min at 37°C to degrade the RNA. The resulting Cy3 and Cy5-labeled cDNAs were paired and mixed together according to the intended experiment and unincorporated nucleotides were removed using a PCR cleaning kit (Qiagen, Valencia, CA). Cleaned probes were concentrated in a SpeedVac (Savant Instrument, Holbrook, NY) for approximately 5 min to a volume of less than 32 μl prior to being used in hybridization to one array.

### Microarray hybridization reactions

The microarray slides were prehybridized by incubation in 5X SSC, 0.1% SDS, 1% BSA at 42°C for 45 to 60 min. For each slide, the labeled cDNA probe was brought to 30.5 μl with the addition of sterile water. A 1.5 μl aliquot of 10 μg/μl polyA was added and the probe was denatured at 95°C for 3 min. An equal amount (32 μl) of pre-warmed 2X hybridization buffer (50% formamide, 10X SSC, 0.2% SDS, [33] was added to the mixture and the probe was pipetted between the pre-hybridized slide and the cover slip (LifterSlip, Erie Scientific Company, Portsmouth, NH). The slide was placed in a hybridization chamber (Corning, New York, NY) and incubated overnight for 16–20 hrs at 42°C. The next day the cover slip was removed and the slide was washed once in 1X SSC, 0.2% SDS prewarmed to 42°C; once in 0.2X SSC, 0.2% SDS at room temperature; and once in 0.1X SSC at room temperature. The washes were conduced with gentle shaking at 100 rpm for 5 min. Slides were subjected to low speed centrifugation for 2 min at 500 rpm to dry.

### Scanning, quantitation, and normalization

The hybridized slides were scanned with a ScanArray Express fluorescent microarray scanner (Perkin Elmer Life Sciences, Boston, MA) and their fluorescence quantified by ScanArray Express software or by GenePix Pro 3.0 (Axon Instruments, Union City, CA). A perl program was written for post analysis processing of the quantitated image files from the Scan Array Express or GenePix Pro3.0. Local background was subtracted from each spot intensity. Spots showing signal intensities below the 95th percentile of the background distribution in the Cy3 or Cy5 channel were filtered out. The ratio of Cy5 mean to Cy3 mean (*r*) was computed and used to adjust the Cy3 values to Cy3 X sqrt(*r*) and the Cy5 values to Cy5/sqrt(*r*). A between-replicate correction was made using an ANOVA model, which equalized average grid or slide intensities between replicates, for Cy3 and Cy5 separately. The ratio of the resulting adjusted intensities of Cy5 to Cy3 was computed for each spot. The coefficient of variation (standard deviation/mean) across replicates was calculated for each spot to evaluate repeatability of the hybridizations.

### High density filter hybridization and selection of weakly expressing cDNAs

High density nylon filters containing 18,432 non-sequenced cDNA clones from the cDNA library Gm-c1007 made from immature cotyledons were spotted using a Qbot by Incyte Genomics. Before use, the filters were washed in0.5% SDS solution that was heated to 60°C, poured over the membrane, and gently agitated for five minutes. This will rid the filter of any residual debris and will result in a cleaner hybridization.

#### Radiolabelling of probe

Total mRNA from developing cotyledons was labeled with ^33^P-dATP in the following manner: RNA in 8 μl water (up to 5 μg, but generally 2 to 3 μg of mRNA) was combined with 4 μl Oligo dT (0.5 μg/ul, 70 μM, Sigma Lot 29H9065). The mixture was heat treated for 10 min at 70°C and chilled on ice before adding the following: 6 μl of 5X first strand buffer (BRL/Life Tech Cat. #18064-014); 1 μl DTT; 1.5 μl each of 10 mM dGTP, dCTP, and dTTP; 1.5 μl reverse transcriptase (200 units/μl, SuperScript II RT from BRL/Life Tech Cat. #18064-014); and 10 μl ^33^P dATP at 10 mCi/ (NEN, 33P Cat#612H04029). After incubation at 37°C for 90 min, the probe was purified by a passage through a Bio-Spin 30 Chromatography Column (Bio-Rad Cat. #732-6006), then stored at 4°C until ready to be denatured and added to the pre-hybridized filter).

#### Prehybridization

The filter was rolled and placed in a hybridization bottle containing 25 ml of pre hybridization solution without formamide [34] and was prehybridized for 3–4 hrs at 65°C in a rotor oven.

#### Hybridization

Adding the probe to the filter: Once the filter was pre-hybridized, the probe was denatured for 10 min at 95°C and then the entire radiolabeled probe was added directly to the prehybridization mixture (in the bottle with the filer). The hybridization was allowed to proceed for 12–18 hrs.

#### Washing

The filters were washed twice in the pre-warmed (50–55°C) low stringency wash solution (2XSSC, 0.5% SDS, 0.1% Na pyrophosphate) for 15 min each. The filters were then washed for about 2 hrs at 55°C in high stringency buffer (0.1XSSC, 0.5% SDS, O.1% sodium pyrophosphate) with gentle shaking.

#### Imaging

Filters were analyzed with a Typhoon 8600 variable mode imager (Amersham Pharmacia Biotech, Inc, Piscataway, NJ) and imaged with the software package Array Vision (Imaging Research Inc., St. Catharines, Ontario, Canada) to correlate spot intensity and filter position. Spots with very low intensity of 1 to 500 were selected at random in order to enrich for cDNAs representing mRNAs of low abundance. Theses clones were reracked into 384-well plates to form library Gm-r1030 and sent for 5' sequencing at the Washington University Genome Center.

### Distribution of materials

Upon request, all novel materials described in this publication will be made available in a timely manner for noncommercial research purposes. The cDNA clones are available from the Biogenetic Services, Brookings, SD or the American Type Culture Collection, Manasas, VA. Microarrays are available on a cost recovery basis by contacting Lila Vodkin, University of Illinois.

## List of abbreviations

PCR polymerase chain reaction

SSC standard saline citrate

SDS sodium dodecyl sulfate

## Authors' contributions

LOV led the unigene and microarray development, coordinated the project, and drafted the manuscript; AK constructed multiple cDNA libraries included in the unigene cDNAs, performed over 18,000 PCR reactions, performed the library normalization by filter screening shown in Figure 1, the array hybridization reactions including that of Fig 4, and drafted protocols; RS led the informatics and sample tracking efforts for array printing, high throughput PCR, cDNA clone reracking, printed arrays, developed and drafted protocols for analysis of array data; SJC initiated the PCR and array hybridization protocols for the project and constructed several cDNA libraries; DOG performed 10,000 PCR reactions, printed arrays, and participated in clone rearraying; RP contributed to protocol development, PCR production, and gel analysis; GZ accumulated the 'choice clone" cDNAs and performed the isoline hybridization analysis shown in Figure 3; MS contributed to informatics and sample tracking and gel analysis of PCR products; MVS performed EST cluster analysis for development of the unigene set and drafted sections of the paper; ES and CS performed EST analysis and clustering for the unigene set; ER coordinated EST processing and informatics; JE constructed multiple cDNA libraries used in selecting the unigene set; RS led the public EST project for library construction and 5' sequencing; AR-H and JCP provided hydroponically grown plant material and RNAs for Figure 4 and constructed a cDNA library; VC and PC constructed multiple cDNA libraries; GG and LL performed annotations of the 27,500 unigene set; JP and PS led or performed the 3' sequencing of the 27,500 unigene clones.
